# Pharmacokinetic Properties of Artemether, Dihydroartemisinin, Lumefantrine, and Quinine in Pregnant Women with Uncomplicated Plasmodium falciparum Malaria in Uganda

**DOI:** 10.1128/AAC.00683-13

**Published:** 2013-10

**Authors:** Joel Tarning, Frank Kloprogge, Mehul Dhorda, Vincent Jullien, Francois Nosten, Nicholas J. White, Philippe J. Guerin, Patrice Piola

**Affiliations:** Centre for Tropical Medicine, Nuffield Department of Medicine, University of Oxford, Oxford, United Kingdoma; Mahidol-Oxford Tropical Medicine Research Unit, Faculty of Tropical Medicine, Mahidol University, Bangkok, Thailandb; WorldWide Antimalarial Resistance Network (WWARN)c; Epicentre, Mbarara, Ugandad; Center for Vaccine Development—Malaria Group, University of Maryland School of Medicine, Baltimore, Maryland, USAe; Université Paris Descartes, INSERM U663, Assistance Publique-Hôpitaux de Paris, Hôpital Saint-Vincent de Paul, Paris, Francef; Shoklo Malaria Research Unit, Mahidol-Oxford Tropical Medicine Research Unit, Faculty of Tropical Medicine, Mahidol University, Mae Sot, Thailandg; Epicentre, Paris, Franceh

## Abstract

Pregnancy alters the pharmacokinetic properties of many drugs used in the treatment of malaria, usually resulting in lower drug exposures. This increases the risks of treatment failure, adverse outcomes for the fetus, and the development of resistance. The pharmacokinetic properties of artemether and its principal metabolite dihydroartemisinin (*n* = 21), quinine (*n* = 21), and lumefantrine (*n* = 26) in pregnant Ugandan women were studied. Lumefantrine pharmacokinetics in a nonpregnant control group (*n* = 17) were also studied. Frequently sampled patient data were evaluated with noncompartmental analysis. No significant correlation was observed between estimated gestational age and artemether, dihydroartemisinin, lumefantrine, or quinine exposures. Artemether/dihydroartemisinin and quinine exposures were generally low in these pregnant women compared to values reported previously for nonpregnant patients. Median day 7 lumefantrine concentrations were 488 (range, 30.7 to 3,550) ng/ml in pregnant women compared to 720 (339 to 2,150) ng/ml in nonpregnant women (*P* = 0.128). There was no statistical difference in total lumefantrine exposure or maximum concentration. More studies with appropriate control groups in larger series are needed to characterize the degree to which pregnant women are underdosed with current antimalarial dosing regimens.

## INTRODUCTION

Approximately 85 million pregnancies occurred in areas with Plasmodium falciparum transmission in 2007 (1). Worldwide mortality rates from malaria were estimated at 660,000 (lower bound, 490,000; upper bound, 836,000) in 2010 (2). In the same year, an estimated 219 million (154 to 289 million) malaria infections occurred (2). Pregnant women are at higher risk of developing severe forms of malaria than are nonpregnant women, and even an asymptomatic infection(s) impairs fetal development. Malaria is an important cause of abortion and stillbirth. The first-line treatment for uncomplicated P. falciparum malaria is artemisinin-based combination therapy (ACT). This comprises an artemisinin-class drug and a more slowly eliminated partner drug (3). Quinine is still used widely, especially in the treatment of severe malaria, despite the proven superiority of artesunate (4, 5). The ACTs used today commonly provide excellent cure rates of above 95% (6–21), but resistance to artemisinin has emerged in South East Asia, resulting in slow parasite clearance times and increased treatment failure rates (22, 23). This will also lead to an increased pressure on the partner drugs, since a greater number of residual parasites need to be eliminated by the slowly eliminated partner drug.

Pregnancy alters the pharmacokinetic properties of many drugs. Decreased gut motility, increased plasma volume and water and fat content, and/or several changes in CYP enzyme and UGT activities during pregnancy lead to altered absorption, distribution, and elimination of antimalarial drugs (24–26). Lower drug exposure levels have been reported for artemether/dihydroartemisinin (27), artesunate/dihydroartemisinin (28), dihydroartemisinin (29), lumefantrine (30), atovaquone (31), and proguanil (31) in pregnant women. However, some antimalarials (e.g., piperaquine [29, 32–34], amodiaquine, and desethylamodiaquine [35, 36]) show drug exposure levels in pregnant women similar to those for the nonpregnant adult patient population. Contradictory results of lower (37, 38), similar (38), and higher (39) exposures have been reported for sulfadoxine and pyrimethamine in pregnant women.

Low cure rates (82%) have been reported for pregnant women in Thailand receiving artemether-lumefantrine (40). However, pregnant women in Uganda showed an adequate clinical response after the same treatment (98.2%). This might be explained by differences in pharmacokinetics, different resistance patterns, or higher levels of background immunity (11). The reported pharmacokinetic properties of intravenous quinine did not show significant differences between pregnant (*n* = 8) and nonpregnant (*n* = 8) women with uncomplicated P. falciparum malaria in a small study from Sudan (41). However, in pregnant women with severe P. falciparum malaria (*n* = 10) (42), a short quinine elimination half-life (11.3 versus 16.0 and 18.2 h) and low apparent volume of distribution (0.96 versus 1.67 and 1.18 liters/kg) was reported compared to previously studied patients with uncomplicated P. falciparum malaria and patients with cerebral malaria, respectively (43). However, the pharmacokinetic properties of oral quinine in pregnant women have not been reported in the published literature.

The aim of this study was to evaluate the pharmacokinetic properties of quinine and artemether-lumefantrine when used for malaria treatment in the second and third trimesters of pregnancy in Uganda.

## MATERIALS AND METHODS

### Study design.

This pharmacokinetic study was nested into a larger efficacy study conducted in the Mbarara National Referral Hospital (MNRH) antenatal clinic (ANC) in Uganda (11). Full clinical details for the pregnant women in that trial are reported elsewhere (11).

The trial was registered at ClinicalTrials.gov (NCT00495508), and ethical approval was obtained from the Uganda National Council for Science and Technology (ethics committee), the Mbarara University Institutional Ethics Committee, Mbarara University Faculty of Medicine Research and Ethics Committee, and the “Comité de Protection des Personnes,” Iles de France XI, France.

Inclusion criteria were residence in the Mbarara Municipality (radius of 15 km from MNRH), an estimated gestation age (EGA) of at least 13 weeks, and P. falciparum mixed infection or monoinfection (detected by microscopy). Exclusion criteria were severe anemia (hemoglobin [Hb], <7 g/dl), known allergy to artemisinin derivatives, lumefantrine, or quinine, a P. falciparum parasitemia level above 250,000 parasites/μl, signs or symptoms of severe malaria requiring parenteral treatment, or inability to comply with the specified follow-up schedule. Patients were enrolled if written informed consent was obtained and if they fulfilled all inclusion criteria and met none of the exclusion criteria. Nonpregnant women in the lumefantrine control group were also enrolled from the efficacy study (up to 1 year during follow-up) and matched to the pregnant women in the lumefantrine arm by history of fever, axillary temperature of >37.5°C, smoking status, and parasitemia levels of <1,000, 1,001 to 25,000, or 25,001 to 250,000 parasites/μl.

### Treatment regimen.

Patients in the artemether/lumefantrine arm were given four tablets of the fixed oral combination of artemether and lumefantrine (Coartem Novartis Pharma AG, Basel, Switzerland; each tablet contained 20 mg artemether and 120 mg lumefantrine) twice daily for 3 days (planned protocol times at 0, 8, 24, 36, 48, and 60 h). Milk tea (200 ml) was given with each dose to optimize the oral bioavailability of lumefantrine (44). Patients in the quinine arm were given 10 mg of oral quinine sulfate/kg of body weight (Remedica, Limassol, Cyprus; each tablet contained 300 mg of quinine sulfate) three times daily for 7 days (planned protocol times at 0, 8, and 16 h). Drug treatments were supervised for both treatment arms.

If the dose was vomited within 30 min, a full replacement dose was given, and if the dose was vomited between 30 min and 1 h, a half replacement dose was given. The patient was withdrawn from the study and treated with rescue treatment if the replacement dose was vomited again within 30 min (i.e., oral quinine for patients in the artemether/lumefantrine arm and oral artemether/lumefantrine for patients in the quinine arm).

### Pharmacokinetic sampling and drug quantification.

Venous blood samples (2 ml) for artemether/dihydroartemisinin measurement were drawn from an indwelling cannula into heparinized tubes at 0, 0.25, 0.5, 0.75, 1, 1.25, 1.5, 1.75, 2, 2.5, 3, 4, 6, 8, and 10 h after the last dose. Blood samples (2 ml) for lumefantrine measurement were collected similarly at 0, 4, 8, 12, 24, 28, 36, 40, 48, 52, 60, 60.5, 61, 62, 64, 66, 68, 72, 84, 108, 132, 156, 180, 204, and 228 h after the first dose. Lumefantrine day 7 samples (168 h) were also drawn from most patients. Blood samples (2 ml) for quinine measurement were collected similarly at 0, 1, 2, 3, 4, 8, 16, 24, 48, 72, 96, 120, 144, 160, 161, 162, 163, 164, 168, 170, 172, 176, and 184 h after the first dose.

Blood samples were centrifuged for 5 min at 1,400 × *g*, and plasma was stored at −70°C or below until analysis. The artemether/dihydroartemisinin and lumefantrine plasma samples were shipped on dry ice to the Department of Clinical Pharmacology, Mahidol-Oxford Tropical Medicine Research Unit, Bangkok, Thailand, and the quinine plasma samples were shipped on dry ice to the Service de Pharmacologie Clinique, Hôpital St Vincent de Paul in Paris, France, for quantification.

Quantification of artemether and dihydroartemisinin was performed by a previously published method using liquid chromatography (LC) coupled to tandem mass spectrometry (MS/MS) (45). Triplicates of quality control samples at three concentrations (3.46 ng/ml, 36 ng/ml, and 375 ng/ml) for both artemether and dihydroartemisinin were analyzed within every batch to ensure precision and accuracy during quantification. The overall relative standard variation (i.e., RSD) was less than 5.4%, and the lower limit of quantification (LLOQ) was set to 1.43 ng/ml for both compounds.

Quantification of lumefantrine was performed by a previously published method using LC with UV detection (46). Triplicates of quality control samples were analyzed at three concentrations (200 ng/ml, 2,000 ng/ml, and 15,000 ng/ml for pregnant patients and 74.3 ng/ml, 1,056 ng/ml, and 15,000 ng/ml for nonpregnant patients). The overall RSD was less than 9.99% for all quality control samples, and the LLOQ was set to 26 ng/ml.

Quinine drug analysis was performed using LC with fluorimetric detection (unpublished method). Fifty microliters of 0.1 M NaOH and 50 μl of the internal standard (hydroquinidine, 7.5 μg/liter) were added to 50 μl of plasma. Liquid/liquid extraction was performed with 4 ml of dichloromethane-isopropyl alcohol (80:20). After 10 min of mixing, the samples were centrifuged and the supernatant was separated and evaporated under a stream of nitrogen. The dry residue was reconstituted with 100 μl of the mobile phase, and 30 μl was injected in the chromatographic system. Chromatographic separation was performed with a Cluzeau C_8_ Plus Satisfaction column (250 by 3 mm; particle size, 3 μm; Sainte Foy la Grande, France) with a mobile phase consisting of 0.1 M dihydrogen potassium phosphate-acetonitrile-acetic acid (695:300:5). The retention times of quinine and the internal standard were 4.9 min and 6.1 min, respectively. Excitation and emission wavelengths were 350 and 440 nm, respectively. The recovery was between 76% and 80% within the calibration range of 1 to 10 μg/ml. Duplicates of quality control samples were analyzed at three concentrations, 2 μg/ml, 6 μg/ml, and 8 μg/ml. Overall accuracy (bias) and precision (RSD) were less than 5.0% and 9.9%, respectively, and the LLOQ was set to 1 μg/ml. Both bioanalytical laboratories participate in the WorldWide Antimalarial Resistance Network (WWARN) quality control and assurance proficiency testing program (http://www.wwarn.org/toolkit/qaqc).

### Pharmacokinetic analysis.

Individual plasma concentration-time data were evaluated using a noncompartmental approach with WinNonlin version 5.3 (Pharsight Corporation, CA). Total exposure (area under the concentration-time curve) from zero time up to the last measured concentration (AUC_0–LAST_) was calculated using the linear trapezoidal method for ascending concentrations and the logarithmic trapezoidal method for declining concentrations. The terminal elimination half-life (*T*_1/2_) was estimated by the slope (λ_Z_) of the best-fit log-linear regression of the observed concentrations in the terminal elimination phase. Drug exposure was extrapolated from the last observed concentration to infinity (*C*_LAST_/λ_Z_) for each individual subject to compute total drug exposure (AUC_0–∞_). The maximum concentration of drug in plasma (*C*_max_), time to maximum concentration of drug in plasma (*T*_max_), and the lag time before quantifiable absorption (*T*_lag_) were taken directly from the observed data. The apparent volume of distribution (apparent volume of distribution in the terminal elimination phase [*V*_Z_]/oral bioavailability [*F*]) and oral clearance (elimination clearance [*CL*]/*F*) were computed individually using (equations 1) and 2.

(1) $$\frac{V_{z}}{F}=\frac{DOSE}{\frac{\ln2\;}{t_{1/2}}\times AUC}$$

(2) $$\frac{CL}{F}=\frac{DOSE}{AUC}$$

Patients who did not provide a sufficient number of samples for a full pharmacokinetic evaluation were excluded from the analysis but included in the summary statistics for *C*_max_, *T*_max_, and *T*_lag_ if the data allowed. Complete *in vivo* conversion of artemether into dihydroartemisinin was assumed, and the administered dose of dihydroartemisinin was calculated using the relative difference in molecular weights. Lumefantrine samples were collected frequently for all doses and could therefore capture the accumulation of drug over time. Residual lumefantrine exposure from the 3 days of dosing could not be accurately subtracted from the lumefantrine exposure of the last dose because of its multicompartment pharmacokinetics and the long terminal elimination half-life. Therefore, the total dose of lumefantrine (i.e., the sum of the six doses) was used as the input dose together with all observed concentration-time data in the noncompartmental analysis of lumefantrine. Quinine plasma samples taken after the first dose (samples taken up to 8 h after the first dose) were used for analysis, since subsequent samples were too sparse (i.e., only one trough value per day) to compensate fully for the accumulation of the drug over time.

Individual pharmacokinetic parameter estimates for lumefantrine were compared between pregnant women and nonpregnant women using the Mann-Whitney test in STATA v.11. Artemether/dihydroartemisinin and quinine pharmacokinetics were compared to literature values.

## RESULTS

### Pharmacodynamics.

Between October 2006 and May 2009, 304 women were recruited in an efficacy trial (152 in the quinine arm and 152 in the artemether-lumefantrine arm). The study participants originated from a cohort of 1,197 pregnant women who were screened for malaria on a weekly basis. The day 42 PCR-adjusted cure rate among analyzable patients was high in both arms: 97.6% (95% confidence interval, 93.1 to 99.5%) in the quinine arm and 99.3% (96.0 to 99.9%) in the artemether-lumefantrine arm. Details have been published elsewhere (11), and admission demographics for the patients included in the pharmacokinetic study are summarized in Table 1.

**Table 1:** Admission demographics of patients included in the pharmacokinetic study

Parameter	Result (range)^*i*^ for:			
	Artemether/dihydroartemisinin	Lumefantrine		Quinine
	Pregnant women (*n* = 21)	Pregnant women (*n* = 26)	Nonpregnant women (*n* = 17)	Pregnant women (*n* = 23)
Age (yr)	21 (16–35)	20 (18–38)	21 (18–29)	21 (18–37)
Body wt (kg)	55 (49–88)	56 (44–74)	49 (40–63)	56 (44–71)
Gestational age (wk)	27 (13–36)	22.5 (16–38)		26 (13–37)
2nd trimester (%)	47.6	69.2		52.2
3rd trimester (%)	52.4	30.8		47.8
Body temp (°C)	36.7 (36.0–38.5)	36.7 (36.0–39.3)	36.7 (36.1–38.2)	37.1 (36.0–38.9)
P. falciparum (parasites/μl)	1,570 (88.0–148,000)	638 (32–11,800)	751 (48–152,190)	2,160 (39–44,500)
Platelets (10^9^/liter)	167 (64–285)	185 (83–255)^*a*^	153 (78–247)^*d*^	132 (15–313)
Bilirubin (mg/dl)	0.91 (0.56–5.53)	0.75 (0.25–2.27)^*b*^	1.41 (0.39–2.80)	1.30 (0.31–3.36)
Hematocrit (%)	34.0 (23.2–44.5)	29.5 (20.3–35.0)^*a*^	37.9 (35.0–43.3)^*d*^	31.3 (22.1–39.8)
Diastolic blood pressure (mm Hg)	60.0 (46.0–75.0)	60.5 (44.0–73.0)	65.0 (49.0–81.0)	63.0 (45.0–80.0)
Hemoglobin (g/dl)	11.3 (7.6–14.6)	10.0 (6.9–12.4)^*a*^	12.8 (11.5–14.5)^*c*^	10.4 (7.4–12.7)
Red blood cells (10^12^/liter)	3.71 (2.37–4.79)	3.39 (2.23–4.51)^*a*^	4.28 (3.89–4.81)^*d*^	3.43 (2.37–4.50)
Neutrophils (10^9^/liter)	2.75 (1.14–4.13)	3.30 (1.89–6.03)^*e*^	2.58 (0.74–4.86)^*g*^	2.47 (0.55–6.53)^*h*^
Eosinophils (10^6^/liter)	70 (20–570)	230 (40–810)^*f*^	280 (110–640)^*g*^	85 (10–300)^*h*^
Basophils (10^6^/liter)	20 (10–60)	20 (10–50)^*e*^	40 (20–160)^*d*^	30 (10–80)
Lymphocytes (10^9^/liter)	1.98 (1.12–3.51)	1.82 (0.77–3.75)^*a*^	1.34 (0.62–2.99)^*d*^	2.21 (0.69–3.61)
Monocytes (10^9^/liter)	0.55 (0.26–1.00)	0.31 (0.01–3.02)^*a*^	0.26 (0.02–0.44)^*d*^	0.63 (0.17–1.34)
ALAT^*j*^ results (IU/liter)	14.0 (5.0–35.0)	16.0 (8.0–86.7)^*b*^	23.0 (7.0–109)	16.0 (8.0–26.0)
Creatinine results (mg/dl)	0.47 (0.33–0.66)	0.54 (0.38–0.93)^*g*^	0.71 (0.40–0.96)	0.49 (0.35–1.29)

a Based on results for 17 patients.

b Based on results for 21 patients.

c Based on results for 16 patients.

d Based on results for 13 patients.

e Based on results for 15 patients.

f Based on results for 14 patients.

g Based on results for 10 patients.

h Based on results for 22 patients.

i Values are given as the median (range) unless otherwise specified.

j ALAT, alanine aminotransferase.

### Artemether and dihydroartemisinin pharmacokinetics.

**Fig 1:**
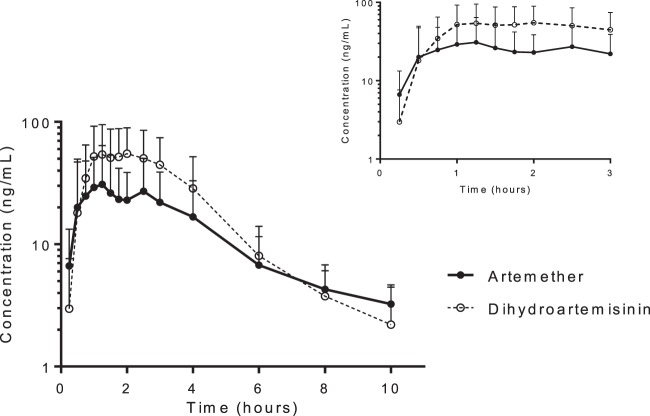
Mean artemether and dihydroartemisinin venous plasma concentration-time curves after the last dose in pregnant women with uncomplicated P. falciparum malaria. Error bars indicate standard deviations. Inset shows concentration-time profiles for up to 3 h after the last dose.

Artemether and dihydroartemisinin pharmacokinetics in pregnant women were well described (*n* = 21) with P. falciparum malaria and pharmacokinetic parameters reported elsewhere (47) (Fig. 1). Several patients showed a clear distribution phase with multicompartment pharmacokinetics, whereas other patients did not. A double absorption peak for both artemether and dihydroartemisinin was observed for 3 patients. One patient had a double absorption peak for artemether only, and one patient had a double peak for dihydroartemisinin only. The second peaks occurred between 2 and 4 h after dosing. No cases of vomiting or additional dosing were recorded. Total median artemether maximum concentration (35.4 [range, 5.69 to 143] ng/ml) and exposure (104 [10.8 to 351] h · ng/ml) and dihydroartemisinin maximum concentration (83.0 [18.8 to 153] ng/ml) and exposure (200 [55.9 to 456] h · ng/ml) displayed substantial between-patient variability (Fig. 1). A regression analysis of total exposure and maximum concentration versus estimated gestational age did not deviate from zero for artemether (*P* = 0.487 and *P* = 0.671, respectively) or dihydroartemisinin (*P* = 0.773 and *P* = 0.866, respectively), which suggests no significant correlation between gestational age and drug exposure (data not shown). Similarly, there was no significant difference between trimesters in total artemether exposure (*P* = 0.972), dihydroartemisinin exposure (*P* = 0.972), maximum artemether concentration (*P* = 0.751), or maximum dihydroartemisinin concentration (*P* = 0.503). The same was seen when combining the total exposures and maximum concentrations of artemether and dihydroartemisinin for total malaria activity (*P* = 0.517 and *P* = 0.682, respectively). Similarly, there was no significant difference between trimesters in combined total exposure (*P* = 0.976) or combined maximum plasma concentration (*P* = 0.689).

### Lumefantrine pharmacokinetics.

**Fig 2:**
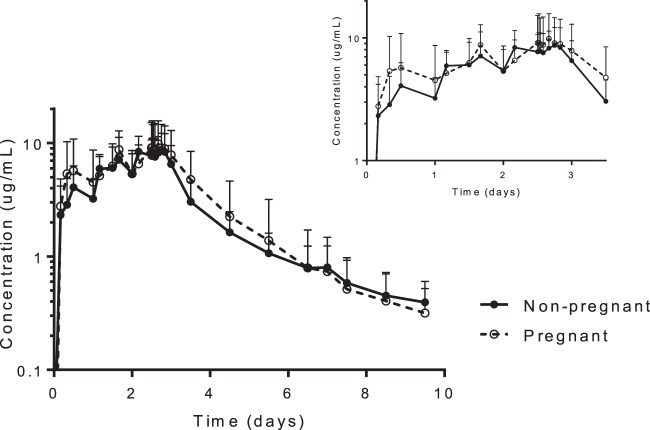
Mean lumefantrine venous plasma concentration-time curves in pregnant and nonpregnant women with uncomplicated P. falciparum malaria. Error bars indicate standard deviations. Inset shows concentration-time profiles for up to 3 days after dose initiation.

Lumefantrine pharmacokinetics in pregnant (*n* = 26) and nonpregnant (*n* = 17) women with P. falciparum malaria were well described (Table 2; Fig. 2). Times to maximum concentration and the terminal elimination half-life estimates were shorter in pregnant than in nonpregnant patients (Table 2). However, there was no statistical difference in total lumefantrine exposure, apparent volume of distribution, or elimination clearance between the two groups. Therefore, a compartmental analysis is needed to evaluate and understand potential differences in the pharmacokinetics between pregnant and nonpregnant women. Total lumefantrine exposures from 72 h (i.e., 12 h after the last dose) until the last sample were similar in pregnant and nonpregnant women (*P* = 0.691). Day 7 concentrations were generally higher in nonpregnant women (median, 720 [range, 339 to 2,150] ng/ml) than in pregnant women (488 [30.7 to 3,550] ng/ml), but this difference did not reach statistical significance (*P* = 0.128). Overall, 5% and 15% of the pregnant women, respectively, had day 7 lumefantrine plasma concentrations below the suggested cutoff values of 175 ng/ml (48) and 280 ng/ml (50) for therapeutic efficacy. However, none of the women in the nonpregnant control group had day 7 lumefantrine plasma concentrations below 280 ng/ml. A regression analysis of total exposure and maximum concentrations versus estimated gestational age did not deviate from zero (*P* = 0.334 and *P* = 0.245, respectively) and suggests no significant correlation between week of gestational age and drug exposure (data not shown). Similarly, there was no significant difference in total exposure (*P* = 0.281) or maximum concentration (*P* = 0.359) between trimesters.

**Table 2:** Noncompartmental analysis of lumefantrine in pregnant and nonpregnant patients with uncomplicated P. falciparum malaria

Parameter^*a*^	Result (range)^*b*^ for:		*P* value
	Pregnant women (*n* = 25)	Nonpregnant women (*n* = 17)	
Total dose (mg/kg)	51.4 (38.9–65.5)^*c*^	58.8 (45.7–72.0)	0.010
*T*_max_ (h)	4.00 (0.0833–12.1)^*c*^	6.00 (1.00–14.0)	0.032
*C*_max_(μg/ml)	9.19 (0.485–22.4)^*c*^	8.88 (4.50–17.0)	0.747
*CL*/*F* (liters/h)	4.40 (1.54–36.3)	4.63 (2.46–9.87)	0.828
*CL*/*F* (liters/h/kg)	0.0829 (0.0288–0.825)	0.0942 (0.0503–0.224)	0.377
*V*/*F* (liters)	414 (63.4–2,510)	421 (227–1,330)	0.450
*V*/*F* (liters/kg)	6.90 (1.22–57.1)	7.65 (4.63–30.3)	0.148
*T*_1/2_ (h)	53.5 (28.5–79.4)	65.7 (48.2–93.7)	0.003
AUC_72–LAST_ (h · μg/ml)	177 (63.0–1,130)	163 (86.1–4,400)	0.691
AUC_72–∞_ (h · μg/ml)	189 (64.7–1,170)	197 (99.0–544)	0.949
AUC_0–LAST_ (h · μg/ml)	632 (77.7–1,840)	591 (270–1,080)	0.729
AUC_0–∞_ (h · μg/ml)	654 (79.4–1,870)	621 (292–1,170)	0.828
AUC_0–∞_/dose (h · μg/ml/[mg/kg])	12.1 (1.21–34.7)	10.6 (4.46–19.9)	0.377
Day 7 concn (ng/ml)	488 (30.7–3,550)^*d*^	720 (339–2,150)	0.128

a *C*_max_, maximum observed plasma concentration after the last dose; *T*_MAX LAST_, observed time after last dose to reach *C*_max_; *CL*, elimination clearance; *V*, apparent volume of distribution; *T_1/2_*, terminal elimination half-life; AUC_72–LAST_, observed area under the plasma concentration-time curve from 72 h to the last observed concentration; AUC_72–∞_, predicted area under the plasma concentration time curve from 72 h to infinity; AUC_0–LAST_, observed area under the plasma concentration-time curve from zero time to the last observed concentration; AUC_0–∞_, predicted area under the plasma concentration time curve from zero time to infinity; Day 7 concn, observed day 7 concentration after repeated drug administration; *F*, oral bioavailability.

b Values are given as the median (range) unless otherwise specified.

c Based on results for 26 patients.

d Based on results for 20 patients.

### Quinine pharmacokinetics.

**Fig 3:**
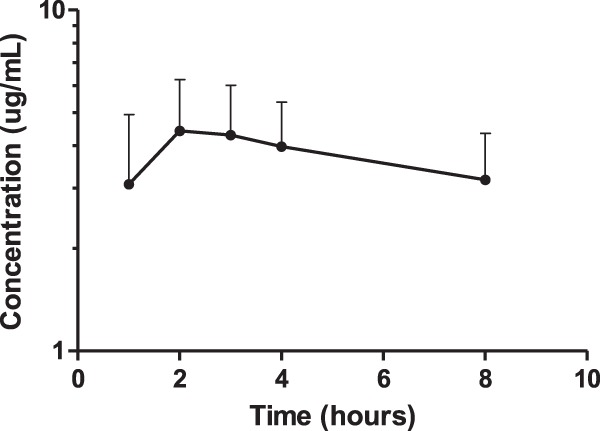
Mean quinine venous plasma concentration-time curve after the first dose in pregnant women with uncomplicated P. falciparum malaria. Error bars indicate standard deviations.

Quinine pharmacokinetics after the first dose were well described in pregnant women (*n* = 21) with P. falciparum malaria (Table 3; Fig. 3). Quinine elimination clearance was approximately 20% higher in pregnant women in this study than in nonpregnant Thai patients (0.11 liters/h/kg versus 0.091 liters/h/kg) (51). This would suggest a lower total exposure in pregnant than in nonpregnant patients. A regression analysis of total exposure and maximum concentration versus estimated gestational age did not deviate from zero (*P* = 0.945 and *P* = 0.375, respectively), which suggests no significant correlation between gestational age and drug exposure (data not shown). Similarly, there was no significant difference in total exposure (*P* = 0.970) or maximum concentration (*P* = 0.433) between trimesters.

**Table 3:** Noncompartmental analysis of quinine in pregnant patients with uncomplicated P. falciparum malaria

Parameter^*a*^	Result for quinine (*n* = 21)^*b*^
Total dose (mg [base]/kg)	7.10 (6.66–7.93)
*C*_max_ (μg/ml)	4.52 (2.58–8.05)
*C*_max_/dose (μg/ml/[mg/kg])	0.640 (0.370–1.20)
*T*_max_ (h)	2.03 (1.07–4.00)
*CL*/*F* (liters/h)	6.07 (1.88–11.3)
*CL*/*F* (liters/h/kg)	0.110 (0.0300–0.210)
*V*/*F* (liters)	74.2 (51.3–161)
*V*/*F* (liters/kg)	1.45 (0.820–2.59)
*T*_1/2_ (h)	9.28 (3.24–21.9)
AUC_0–LAST_ (h · μg/ml)	26.5 (15.2–53.3)
AUC_0–∞_ (h · μg/ml)	61.4 (33.0–231)
AUC_0–∞_/dose (h · μg/ml/[mg/kg])	9.06 (4.65–34.6)
Day 7 concn (μg/ml)^*c*^	3.93 (1.02–7.77)

a *C*_max_, maximum observed plasma concentration after the first dose; *T*_max_, observed time to reach *C*_max_; *CL*, elimination clearance; *V*, apparent volume of distribution; *T*_1/2,_ terminal elimination half-life; AUC_0–LAST_, observed area under the plasma concentration-time curve after the first dose from zero time to the last observed concentration; AUC_0–∞_, predicted area under the plasma concentration time curve after the first dose from zero time to infinity; Day 7 concn, observed day 7 concentration after repeated drug administration; *F*, oral bioavailability.

b Values are given as the median (range) unless otherwise specified.

c Based on results for 23 individuals; day 7 concentrations from individual 199 and 251 were also included.

## DISCUSSION

### Artemether and dihydroartemisinin pharmacokinetics.

Pharmacokinetic parameter estimates in this study were generally comparable to those reported previously for pregnant Thai patients (27), which is the only available comparator group in the literature. Median maximum artemether concentrations and total artemether exposures reported in this study were 35.4 (range, 5.69 to 143) ng/ml and 104 (10.8 to 351) h · ng/ml compared with 35 (14 to 104) ng/ml and 65.6 (10.5 to 280) h · ng/ml, respectively, reported previously for pregnant Thai patients (27). Maximum dihydroartemisinin concentrations and total dihydroartemisinin exposures reported in this study were also in a range similar to that for pregnant Thai patients (*C*_max_, 83.0 [range, 18.8 to 153] ng/ml versus 165 [72 to 224] ng/ml; AUC, 200 [55.9 to 456] h · ng/ml versus 357 [29.8 to 585] h · ng/ml) (27). However, pharmacokinetic parameter estimates vary substantially between different studies which complicate the interpretation of these data, as no nonpregnant contemporaneous control group was available. Total exposure of artemether and dihydroartemisinin was substantially lower than that reported in two nonpregnant patient studies in Thailand (52, 53). Artemether is metabolized by the cytochrome P450 (CYP) enzyme 3A4 into its active metabolite, dihydroartemisinin (54), which is then glucuronidated by UDP-glucuronosyltransferase (UGT) 1A9 and 2B7 (55). Both of these enzyme systems have been reported to be induced during pregnancy (56, 57) and might explain the low exposures in pregnant women compared to literature values. An expansion of the volume of distribution seen in pregnant women could lead to a reduction in peak levels. Although this should not result in a difference in total drug exposure, it might reduce the exposure to concentrations providing maximum effects (i.e., exceeding the minimum parasiticidal concentration). However, only limited data were available in the literature, and larger studies are urgently needed to assess the impact of pregnancy on the pharmacokinetics of artemether and dihydroartemisinin. A more extensive pharmacometric modeling approach based on these data is published elsewhere (47).

### Lumefantrine pharmacokinetics.

Lumefantrine is metabolized predominantly by CYP3A4 (58, 59) and lumefantrine exposure would be expected to be lower in pregnant women than in nonpregnant women. However, there were no statistical differences in total exposure or maximum concentration in pregnant women compared to nonpregnant women in this study. Pharmacokinetic parameter estimates for pregnant and nonpregnant women in this study were also similar to those reported for nonpregnant and pregnant women in the literature (13, 27, 30, 50, 60, 61). Interestingly, the terminal elimination half-life was shorter in pregnant women than in nonpregnant women, which resulted in a substantial, but nonsignificant, difference in measured day 7 concentrations. This might have clinical implications in the duration of posttreatment prophylactic effect and for intermittent preventive treatment in pregnant women. Indeed, 5% and 15% of pregnant women and none of the nonpregnant women in this study had day 7 lumefantrine plasma concentrations below the previously defined therapeutic cutoffs of 175 ng/ml (48) and 280 ng/ml (50), respectively. Furthermore, 31% of the pregnant women in the efficacy study had plasma lumefantrine concentrations below 280 ng/ml at day 7, supporting the suggestion that pregnant women are underdosed (11). The difference between study results (15% versus 31%) might reflect a difference in study size. The relatively low patient numbers in this study and the large interindividual differences might mask potential pregnancy-related differences. A pharmacometric approach could be more informative as it would have greater statistical power to detect true differences.

### Quinine pharmacokinetics.

Quinine is metabolized mainly to its major metabolite, 3-hydroxquinine, by CYP3A4 (62). Pregnancy could theoretically have an impact on the pharmacokinetics of quinine. However, previous studies have reported similar pharmacokinetic properties of quinine in pregnant and nonpregnant patients after parenteral administration of quinine (41, 63). Only sparse literature data are available after oral administration of quinine in nonpregnant patients (51) and no published information is available for that in pregnant women. Total exposure was not reported by Supanaranond et al., but oral clearance (*n* = 15) was somewhat lower in those nonpregnant women than that estimated for the pregnant women in this study (0.091 liters/h/kg versus 0.11 liters/h/kg, respectively). This suggests a decreased exposure in pregnant women compared to nonpregnant adult patients. However, the regression analysis showed no significant correlation between estimated gestational age and exposure parameters, which at least supports a lack of a pregnancy-related effect on quinine pharmacokinetics from the second to the third trimester. A noncompartmental analysis of the data could be performed only after the first dose to avoid the accumulation of drug over time, and a pharmacometric approach might therefore be more appropriate in order to utilize all the available data. This methodology could give more insight about the impact of gestational age, disease, and other relevant biological covariates. Studies of pregnant and nonpregnant women with uncomplicated malaria are needed.

The impact of pharmacokinetic changes on therapeutic responses will be greatest in nonimmune mothers. In many parts of Uganda, malaria transmission is intense and host immune responses can eliminate partially treated infections. Failure rates with artemether-lumefantrine in pregnant women studied in Thailand were 10 times higher than in those in Uganda, despite similar dose regimens and relatively similar drug exposures.

In conclusion, pharmacokinetics of artemether/dihydroartemisinin, lumefantrine, and quinine were well characterized in pregnant patients with uncomplicated P. falciparum malaria. Lumefantrine pharmacokinetics was also evaluated in a nonpregnant control group and resulted in no statistical difference in total exposure between the groups. However, the terminal elimination half-life was shorter in pregnant women than in nonpregnant women, which will affect cure rates and postprophylactic effects, particularly in women with little background immunity. Artemether/dihydroartemisinin and quinine exposures were generally low in pregnant women compared to literature data, but more data are needed to evaluate the potential impact of pregnancy on therapeutic responses.
